# In-Situ Measurement and Slow-Tool-Servo Compensation Method of Roundness Error of a Precision Mandrel

**DOI:** 10.3390/ma15228037

**Published:** 2022-11-14

**Authors:** Zheng Qiao, Yangong Wu, Wentao Chen, Yuanyuan Jia, Bo Wang

**Affiliations:** Center for Precision Engineering, Harbin Institute of Technology, Harbin 150001, China

**Keywords:** roundness error, in situ measurement, compensation, slow tool servo (STS), diamond turning machine, spindle, precision mandrel

## Abstract

This paper describes a method for measuring and compensating the roundness error of a larger mandrel manufactured by an ultra-precision diamond-turning lathe aimed to obtain a calibration cylinder with a roundness of less than 0.1 μm. The diamond-turning machine has a cross-stacked hydrostatic guideway, produces a cutting depth and feed movement direction, and a dual-spindle system that is firmly connected to the bed. Due to the good repeatability of aerostatic spindles, only in situ rather than online real-time measurements are required. To this end, three high-precision capacitance displacement sensors were utilized to detect the cross-section of the workpiece and the time domain via the three-point error separation technique to separate the roundness error from the rotation motion error. The slow tool servo (STS) cutting technique was employed to compensate for the roundness error, which did not require extra axes, only the excellent dynamic response of the feed axis; hence, the servo control parameters could be suitably adjusted. The experimental results reveal that the low-order harmonic error, often caused by aerostatic spindles, is almost removed completely. For this particular lathe, the experiments indicate that about 60% of the rotational error motion is compensated, and the roundness error is reduced to less than 0.1 μm, which is evaluated by the least-squares circle method.

## 1. Introduction

The demands for high-precision machining optical parts [1] and advanced mechanical parts [2,3] with sub-micrometer geometric accuracy and nanometer-level surface roughness are increasing. Precision mandrels have many applications in industrial production, for example, the calibration of precision cylindricity test instruments. The roundness of a mandrel finished by precision grinding can be less than 0.5 μm, and that by ultra-precision grinding can be within 0.2 μm [4,5]. However, it is so challenging to achieve a roundness of fewer than 0.1 μm. Commercial small single-point diamond lathes can manufacture mandrels with a roundness smaller than 0.1 μm, but the mandrel size is limited by the bearing capacity of the air spindle and the movement of the guideways [6]. The large single-point diamond lathe self-produced by the authors can process precision mandrels with diameters and lengths over 0.45 m and 1 m, respectively. However, the workpiece size and absolute accuracy are contradictory, and it is not easy to control the geometric accuracy to the 0.1-micron level since there exist geometric errors in machine tools, thermal errors, and cutting-force-induced errors. To obtain workpieces with extremely high roundness, appropriate error compensation methods must be adopted [7].

The geometric errors are those in a machine that exists due to its initial design, the inaccuracies built-in during assembly, and as a result of the components exploited in the machine. The kinematic errors are concerned with the relative motion errors of several moving machine components that must move per precise functional requirements [8]. For a typical ultra-precision three-axis lathe, geometric errors have various components, such as linear displacement error (positioning accuracy), straightness, rotary motion error, and squareness error. The linear interpolation isometric compensation eliminates most of the linear movement error and achieves a high positioning accuracy of less than one micrometer in one-meter length. The compensation procedures for straightness, squareness, and kinematic errors have been examined a lot [9,10,11], including constructing a kinematics error model, such as the multi-body theory [12] or a linear motion error model [13], selecting a consistent approach, such as the reversal method [14], and spherical test approach [15] to draw the error map, generate compensation data according to the machining path, and modify the compensation data according to the machining results. From the view of the control theory and mechanical dynamics, the basis for implementing the above procedure is the axis that moves the compensation data and can quickly respond during the total machining process. For instance, compensation for the squareness error in a typical lathe requires the x-axis to move only less than 50 μm, while the z-axis moves at a very low speed over a distance of one meter.

However, the problem arises when it comes to compensating for the spindle movement error and any other errors involved in the spindle movement error since an axis that could quickly move back and forth is required. The rotating speed of a rotary spindle is generally higher than 300 rpm, and the linear axes in a generally designed machine could not effectively track the position command of this high frequency. Therefore, a fast-tool-servo (FTS) unit mounted on an X-slide is more commonly exploited. The large optics diamond turning machine (LODTM) project was the earliest case that successfully applied the FTS to track the high-frequency and small-amplitude spindle motion [16,17] and was the beginning of piezoelectric (PZT) as an actuator and capacitance displacement probe to the feedback component [18]. Typically, an FTS unit has a bandwidth between 100 Hz and 3 kHz, a stroke from 1 mm to 10 μm, and a resolution that can reach less than 1 nm. Since then, many researchers have utilized an FTS to compensate for rotating errors during the precision turning of shafts. Chen and Yang [19] developed a PZT actuator and a compensatory tool holder for the online compensation of spindle error motion. The dynamic compensation test on a CG6125 precision lathe revealed that the roundness error of the workpiece could be lessened to 0.4 μm. Xu et al. [20] attempted to refine a cutting tool system for active error compensation by employing the ultrasonic vibration cutting technique. Two PZT actuators are integrated into the cutting tool, one of which is for active error compensation and is satisfactory in terms of the improved roundness of the machined workpieces. Fung et al. [21,22,23] employed forecasting and compensatory control (FCC) to enhance the workpiece roundness accuracy in taper turning on a test lathe, and an improvement of 24–38% for the roundness error was achieved. Gao et al. [24] incorporated a PZT actuator in the boring bar servo to achieve online compensation and tried to reduce the machining errors of a small overhung boring bar by real-time error compensation. The obtained results of cutting tests demonstrated that an improvement of 40% in the roundness errors could be achieved. Hong et al. [25] proposed a compensation approach to accordingly modify the FTS path for each component error to pre-compensate the induced profile errors. The typical micro-structured surfaces, such as sine-like waves along the radial direction and micro-lens array, were machined to display the effect of the component errors on the profile accuracy.

The above-mentioned research works on spindle error compensation have been carried out by employing online measurement and synchronous compensation. The signal of the error motion requires hardware support that can be collected and controlled at high speeds, and chips or cutting fluid may cause lots of noise by affecting the signal collection process. For ceramic bearing or hydrostatic spindles, the online measurement of errors is necessary. For the ceramic ball bearing spindles, small synchronous errors and large asynchronous errors are commonly detectable, while the hydrostatic spindle exhibits a double-cycle phenomenon, and both lead to poor error repeatability during every cycle of the spindle rotation. However, the air-bearing spindle shows extremely high repeatability, whose asynchronous error is often less than 5 nm, and therefore, online measurement is not required. In situ measurements significantly lessen the collection speed requirement and do not require special protection for the acquisition system. Li et al. [26,27] adopted an in situ error measurement system and an error separating technique to assess the error distribution of an aerostatic bearing and exploit a PZT micro-positioning system, which is an FTS system, in essence, to demonstrate the reduction in the roundness error by more than an order of magnitude.

The above literature shows that most researchers tend to use an online measurement and FTS to compensate for the roundness error, which requires very complex measurement and control systems. In the present paper, a systematic compensation procedure with the aim of improving the roundness in ultra-precision turning is introduced, which includes two basic steps: in situ measurement and slow tool servo (STS) compensation. An in situ measurement system is integrated into the machine to distinguish the spindle radial error motion (SREM) and the workpiece’s roundness error (WRE), which greatly eliminates random factors compared with online measurements. With the help of some existing STS experience in our research group, the compensation process did not require an extra and expensive FTS system, meaning that the machine could fully utilize the capabilities without the need for additional micro-motion systems. Finally, the machining of the workpieces with a roundness of fewer than 0.1 μm was realized and discussed.

## 2. In Situ Measurement of Roundness Error in a Diamond Turning Machine

### 2.1. The Machine Tool and Roundness Measurement System

**Figure 1:**
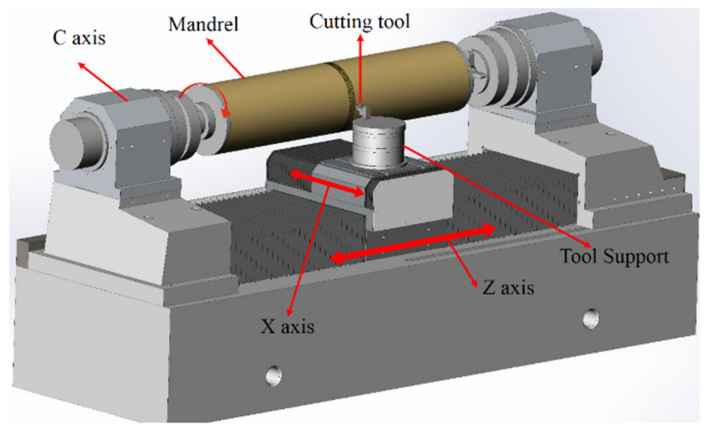
Structure layout of the mandrel ultra-precision machine tool.

The mechanical structure layout of the mandrel ultra-precision machine tool has been presented in Figure 1. This ultra-precision machine tool adopts the XZC three-axis linkage scheme, in which the rotation of the spindle and X Z-slide are synchronized by an NC controller. The X and Z axes represent the linear motion perpendicular to and parallel to the major axis of the mandrel, respectively. The position of the X-slide is measured by a linear encoder with a 0.008 nm resolution. The maximum portable range of the X-slide is 250 mm. The spindle is equipped with a rotary encoder of 360,000 pulses/revolution for positioning. The C-axis can perform both precise angular displacement movements and high-speed rotation as the main shaft. If the size and mass of the mandrel are large, it is clamped at both ends, so this precision machine tool is equipped with two sets of spindle systems. This article discusses the roundness compensation method, so only the headstock spindle is exploited to clamp the smaller workpiece.

The spindle system of this ultra-precision machine tool is supported by an orifice-type aerostatic bearing. By precisely evaluating its design factors and appropriately raising the diameter of the rotor to grow the radial bearing area, the spindle can meet the requirements of a high bearing capacity, as well as high stiffness. The aerostatic lubrication makes the main shaft have a high rotation accuracy, extremely low friction coefficient, and low speed without creep [28]. By optimizing the selection of a larger air film thickness, the temperature increase due to the air film shearing is reduced. As a result, the machine tool can quickly reach thermal equilibrium at various speeds, and the influence of the machine tool’s thermal errors on machining accuracy is decreased to a very low level.

**Figure 2:**
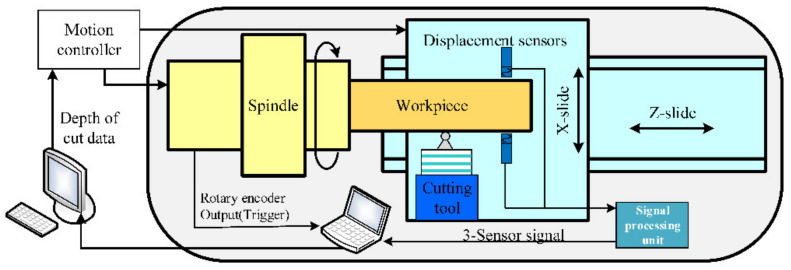
Schematic representation of the measurement and compensation system.

**Figure 3:**
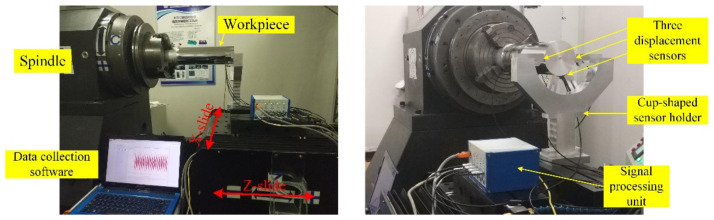
Photographs of the diamond turning machine and measuring devices: (**left**) front view, (**right**) side view.

Figure 2 presents a schematic representation of the measurement and compensation system. Note that although the measuring system and cutting system are given in the same schematic diagram, they are not simultaneously implemented. Figure 3 illustrates a photograph of the diamond turning machine and the experimental setup exploited for the measurement. For ultra-precision machining, the magnitudes of the WRE and SREM are of the same order. According to the error separating principle, an in situ roundness measuring system by the 3-sensor approach is built up.

As can be seen in Figure 2 and Figure 3, the measurement process of the roundness error is as follows: three capacitive displacement sensors are fixed on the cup-shaped mounting holder. These three probes are radially mounted along the same measured section of the mandrel, and their spatial position is guaranteed by the machining accuracy of the cup-shaped holder. It is worth mentioning that from the compensation point of view, it would be better to mount one sensor on the position of the diamond tool so that the measurement position could be matched to the cutting point. In the setup illustrated in Figure 3, the cutting point on the workpiece surface is slightly different from the measurement one, whose spatial position change does not exceed 0.1 mm, ensured by the trial-cutting method. Since the mandrel radius is much larger than the spatial position change, the position discrepancy could be rationally ignored in the experiment. The spindle drives the workpiece to rotate clockwise at a constant speed of 300 rpm, and the sensor readings are obtained after the rotating speed becomes stable. During data acquisition, the synchronism of the workpiece rotation angle and sensor reading is ensured through a grating trigger signal.

The displacement sensors chosen for the measurement are the Micro-Epsilon capacitive sensor capaNCDT CS02, with a range of 200 μm, a resolution of 4 nm, a maximum sampling rate of 5 kHz, and a linearity of less than 0.2% full-scale output (FSO). Since the mandrel is fine by a single-crystal diamond tool, the actual measurement range is usually within 2 μm. The linearity of those probes measuring a cylinder with a nominal diameter of 100 mm has been calibrated. The linearity of the capacitive displacement sensor in small-scale measurement is better than the full-scale linearity, so the measurement accuracy of a single probe can be controlled to about 4 nm.

### 2.2. The Principle of Multiprobe Error Separation

When measuring the outer contour of the mandrel, the data received by the sensors not only includes the cross-sectional contour signal but also the SREM. In the present paper, the time-domain three-point methodology is employed to distinguish the SREM. This approach does not require performing two Fourier transforms, such as the traditional frequency-domain three-point method, which simplifies the calculation process and substantially enhances the calculation efficiency [29].

**Figure 4:**
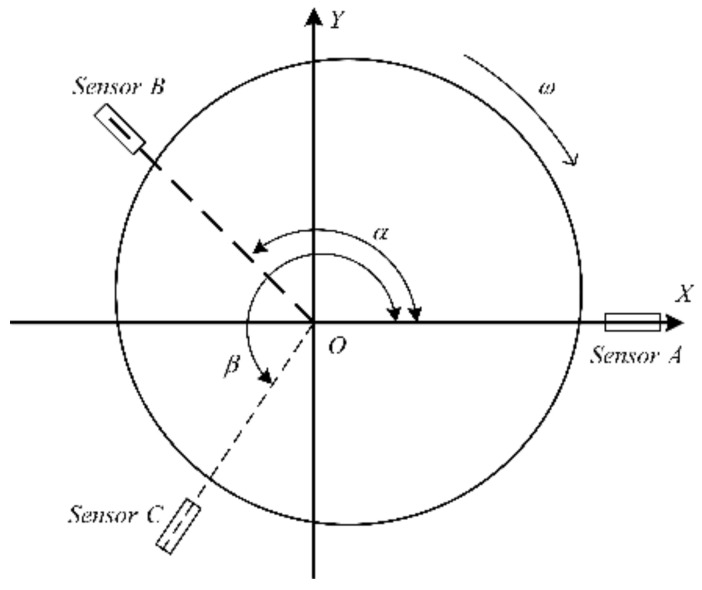
Schematic view of the three-probe error separation method.

Figure 4 illustrates the schematic representation of the three-probe error separation method. The displacement sensors are installed radially along the measured section, and the installation angles of sensors A, B, and C are set as 0, α, and β, respectively (α= 99° 50″, β= 202° 30″). A rectangular coordinate system XOY is established with the intersection O of the axes of sensors A, B, and C as the origin. The total number of sampling points is denoted by N when the spindle rotates one revolution at a constant speed θ = ωt, where t is the time parameter, and ω represents the angular velocity of the spindle. The outputs of the three sensors, i.e., $m_{A}\left(i\right)$, $m_{B}\left(i\right)$, and $m_{C}\left(i\right)$, can be expressed by:

(1) $$\left\{\begin{array}{c} m_{A}\left(i\right)=s\left(i\right)+\delta_{x}\left(i\right)\;\;\;\;\;\;\;\;\;\;\;\;\;\;\\ m_{B}\left(i\right)=s\left(i+k_{1}\right)+\delta_{x}\left(i\right)\cos\frac{2\pi k_{1}}{N}+\delta_{y}\left(i\right)\sin\frac{2\pi k_{1}}{N}\\ m_{C}\left(i\right)=s\left(i+k_{2}\right)+\delta_{x}\left(i\right)\cos\frac{2\pi k_{2}}{N}+\delta_{y}\left(i\right)\sin\frac{2\pi k_{2}}{N} \end{array}\right.$$

where $\delta_{x}\left(i\right)$ and $\delta_{y}\left(i\right)$ represent the SREM values in the direction of *OX* and *OY*, $k_{1}$ and $k_{2}$ denote the measurement intervals of sensors *B* and *C* with respect to sensor *A*, as such that $k_{1}=\frac{\alpha N}{2\pi },k_{2}=\frac{\beta N}{2\pi }$, *s*(*i*) is the discrete profile data of the WRE, *i* represents the *i-*th sensor sampling point (i=0, 1, 2,…,N−1). By shifting the readings of sensor *A* by $k_{1}$ and $k_{2}$, and then subtracting them from the readings of sensors *B* and *C*, we can arrive at the following equations:

(2) $$\{\begin{array}{c} m_{B}\left(i\right)-m_{A}\left(i+k_{1}\right)=\delta_{x}\left(i\right)\cos\frac{2\pi k_{1}}{N}+\delta_{y}\left(i\right)\sin\frac{2\pi k_{1}}{N}-\delta_{x}\left(i+k_{1}\right)\;\\ m_{C}\left(i\right)-m_{A}\left(i+k_{2}\right)=\delta_{x}\left(i\right)\cos\frac{2\pi k_{2}}{N}+\delta_{y}\left(i\right)\sin\frac{2\pi k_{2}}{N}-\delta_{x}\left(i+k_{2}\right)\; \end{array}$$

The expressions on the left side of Equation (2) are the known quantities, and the right side expressions have only been stated in terms of the SREM values; therefore, by solving Equation (2), we can realize the discrepancies between the SREM and the WRE. The discrete form of Equation (2) in a more compact form is stated by:

(3) $$M=E_{d}D$$

where the vectors M and D are given as,

(4) $$D={\left[\delta_{x}\left(i\right),\delta_{y}\left(i\right)\right]}^{T}$$

(5) $$\begin{array}{c} M={\left[m_{B}\left(i\right)-m_{A}\left(i+k_{1}\right),m_{C}\left(i\right)-m_{A}\left(i+k_{2}\right)\right]}^{T} \end{array}$$

where $E_{d}={\left\{A_{pq}\right\}}_{2\times 2}$ denotes the coefficient matrix, and $A_{pq}\left(p,q=1,2\right)$ is an *N×N* matrix:

(6) $$\begin{array}{c} A_{pq}={\left\{a_{st}\right\}}_{N\times N}=\left\{\begin{array}{c} \cos\frac{2\pi k_{q}}{N}\;s=t\;\;\;\;\;\;\;\;\;\\ -1\;\;\;\;t-s=k_{q}\;\left(s<t\right)\;\;\;\\ -1\;\;\;t-s=k_{q}-N\;\left(s>t\right)\\ 0\;\;\;\;\mathrm{otherwise}\;\;\;\;\;\;\;\; \end{array}\right.\; \end{array}$$

By solving the linear set of equations in Equation (3) for the unknowns, the discrete vectors of the SREM are obtained as follows:

(7) $$D=E_{d}{}^{-1}M$$

A solution to Equation (7) is the SREM at the measured section, and the profile data of the WRE can be derived by substituting it into Equation (1). The profile data of WRE, s(i), only reflects the error variation, so a radius value R should be added when reconstructing the cross-sectional shape in order to make the cross-sectional shape more realistic. The coordinates of each point of the WRE are then calculated by:

(8) $$\left\{\begin{array}{c} x_{i}=\left[R+s\left(i\right)\right]\cos\frac{2\pi i}{N}\\ y_{i}=\left[R+s\left(i\right)\right]\sin\frac{2\pi i}{N} \end{array}\right.$$

After reconstructing the cross-sectional shape of the mandrel, the roundness error can be evaluated.

**Figure 5:**
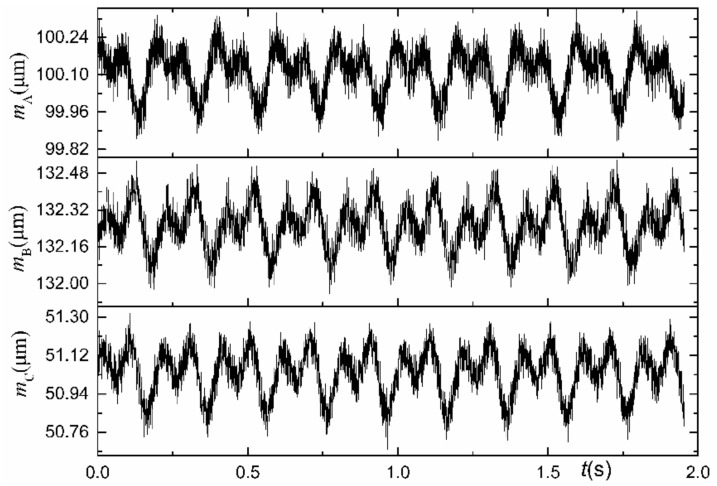
Example of the raw output signals of the displacement-based sensors.

The original signals from the displacement sensors have been presented in Figure 5. In this paper, 128 sample points are equally divided by separating the SREM from the WRE, namely N = 128. In order to reduce the error caused by aliasing, the sampling frequency should be increased. The spindle speed used in the experiment is 300 rpm, and the sampling frequency of the data acquisition system is set equal to fs = 1024 Hz; therefore, the actual sampling number in one rotation period would be N0 = 204.8.

**Figure 6:**
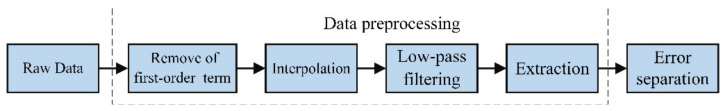
The flowchart of data preprocessing.

**Figure 7:**
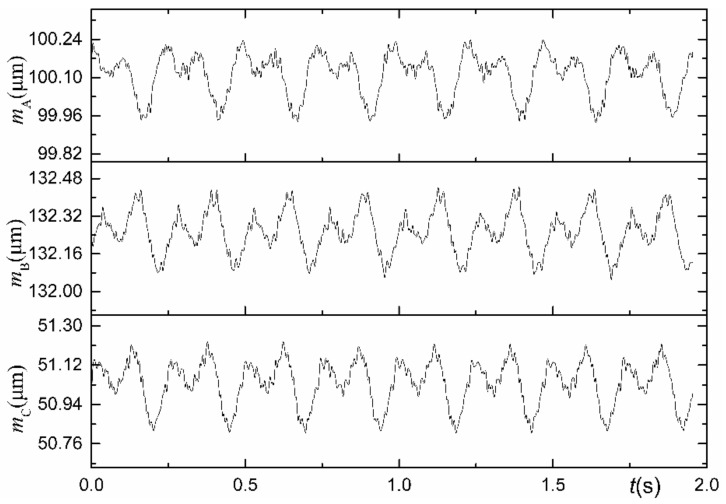
The preprocessed signals of the three probes.

The output signals of the displacement sensors are the synthesis of the SREM and WRE. In the actual measurement illustrated in Figure 5, the output signal also contains DC signal components and noise due to various interferences; therefore, an appropriate data preprocessing must be performed [30]. The data preprocessing flowchart has been provided in Figure 6: the first-order term is removed first, and the required N-point data are obtained through interpolation from the N0-point data, and then low-pass filtering and signal extracting on these data are performed, respectively. The 30th-order zero-phase low-pass filter is selected, which solves the phase distortion problem caused by traditional low-pass filtering while removing the higher-order signal components. The signal of the A sensor after interpolation, filtration, and extraction has been demonstrated in Figure 7.

### 2.3. Roundness Error Evaluation Approach

**Figure 8:**
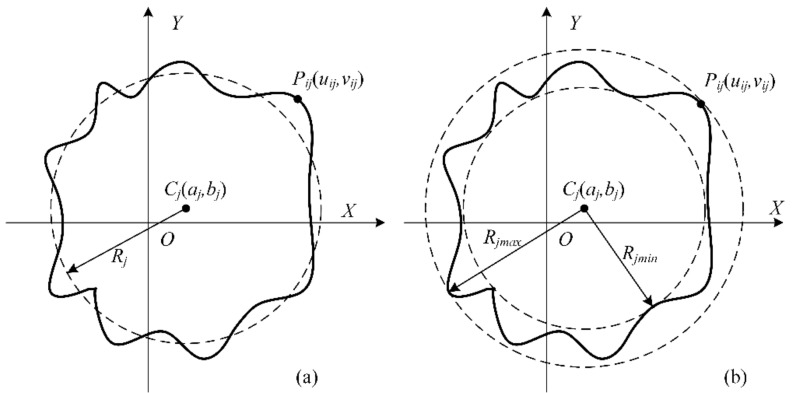
Calculation method of the circle center in roundness error evaluation: (**a**) least-squares circle (LSC), (**b**) minimum zone circle (MZC).

There are four commonly used evaluation methods for assessing the roundness error, namely, the minimum zone circle (MZC) method, the least-squares circle (LSC) method, the maximum inscribed circle (MIC) method, and minimum circumscribed circle (MCC) method. Among them, the MZC method outputs the smallest roundness evaluation value and is the only method that conforms to the standard definition (see Figure 8b). Furthermore, the roundness value evaluated by the LSC is unique, and its calculation method is relatively simple (see Figure 8a).

Let the coordinate values of the *N* measured points be P($x_{i}$,$\;y_{i}$) (i = 0, 1, 2,…, *N* − 1), the circle’s center position is represented by C(*a,b*), and the radius is denoted by *R*. The least-squares circle refers to the circle with the smallest sum of distance squares of its center from the *N* points. Therefore, the objective function, *F*(*a,b,R*), is given by:

(9) $$\begin{array}{c} \begin{array}{c} F\left(a,b,R\right)=\overset{N-1}{\underset{i=0}{\sum }}{\left(R_{i}-R\right)}^{2}=\overset{N-1}{\underset{i=0}{\sum }}{\left(\sqrt{{\left(x_{i}-a\right)}^{2}+{\left(y_{i}-b\right)}^{2}}-R\right)}^{2}, \end{array} \end{array}$$

through solving Equation (9) based on the extreme value theorem, it is obtainable:

(10) $$\left\{\begin{array}{c} a=\frac{2}{N}\overset{N-1}{\underset{i=0}{\sum }}x_{i}\;\;\;\;\;\\ b=\frac{2}{N}\overset{N-1}{\underset{i=0}{\sum }}y_{i}\;\;\;\;\;\\ R=\frac{1}{N}\overset{N-1}{\underset{i=0}{\sum }}\sqrt{x_{i}{}^{2}+y_{i}{}^{2}}\; \end{array}\right.$$

Using Equation (10), the center C(a,b) and radius R of the least-squares circle can be obtained; hence, the roundness error could be evaluated as follows:

(11) $$\begin{array}{c} f_{LSC}=\max\left(\sqrt{{\left(x_{i}-a\right)}^{2}+{\left(y_{i}-b\right)}^{2}}\right)-\min\left(\sqrt{{\left(x_{i}-a\right)}^{2}+{\left(y_{i}-b\right)}^{2}}\right)\; \end{array}$$

## 3. Compensation of Error Motions in the Diamond Turning Machine

### 3.1. Compensation Data Processing Method

**Figure 9:**
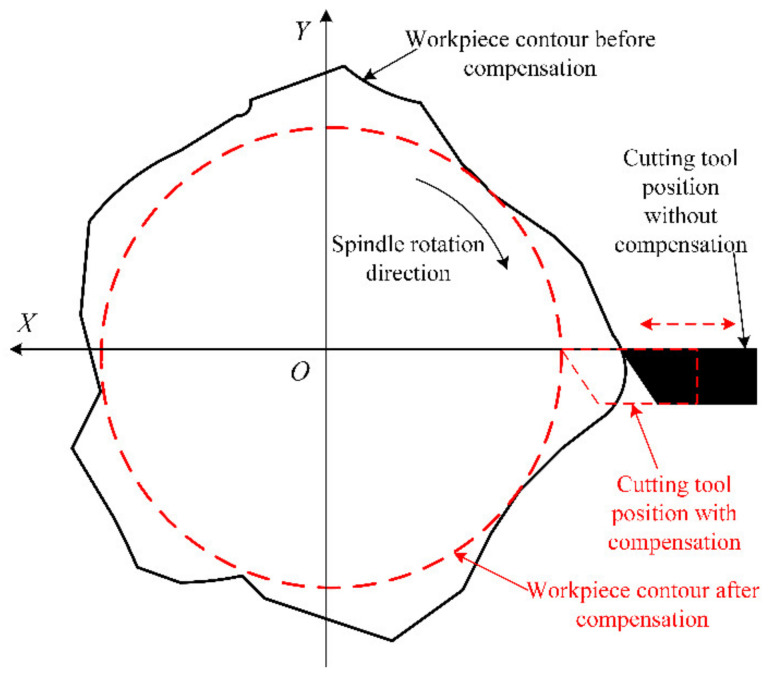
Tool path comparison before and after compensation.

The roundness error of the mandrel is the sum of the ideal circle, machining error, and random error, which can be expressed as:

S(θ) = N(θ) + E(θ) + ε(θ)(12)

where S(θ) represents the actual contour, N(θ) denotes the ideal contour, E(θ) is the machining error, and ε(θ) is the random error, for example, the cutting vibration caused by hard points in the material. Machining is performed by the method of reverse compensation, that is, the tool is offset along the theoretical path by −E(θ), thereby reducing the final actual contour error. In the present work, the error compensation is performed by the STS, and a comparison of the paths before and after tool compensation has been schematically illustrated in Figure 9.

**Figure 10:**
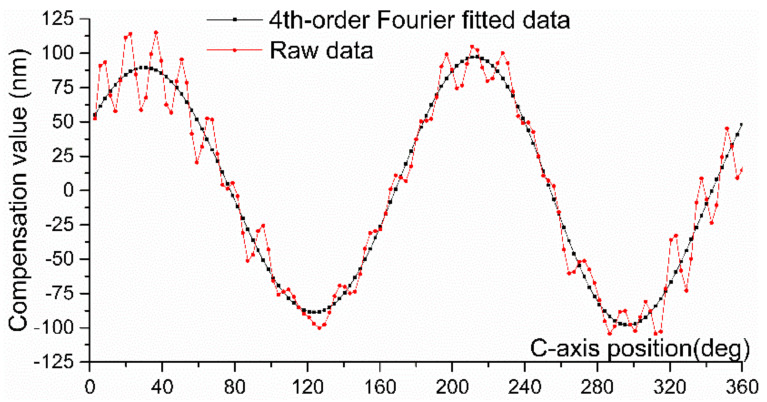
The fitted curve of the WRE compensation data.

The tool path is one of the keys to compensating for the mandrel roundness errors through the STS technique. During STS turning, the C, X, and Z axes of the ultra-precision lathe move together, so it is highly required for a better dynamic response capability of the feed axis. In the present paper, the compensation data are the reverse of the roundness error, which is a series of discrete points. To suppress the dynamic response requirements of the feed axis, the tool path should have good smoothness and continuity. Considering the periodicity of the roundness error, the fourth-order Fourier fitting function is employed to connect those points, and the actual tool path is determined according to the fitted data, E(θ), which has been presented in Figure 10. 

During machining, the tool moves along a curved trajectory relative to the mandrel surface, and the positional relationship of the three axes is:

(13) $$\left\{\begin{array}{c} C_{\mathrm{sts}}=\theta \left(t\right)\;\\ X_{\mathrm{sts}}=-E(\theta )\\ Z_{\mathrm{sts}}=f\cdot t\; \end{array}\right.$$

where E(θ) represents the generated error compensation data, *f* denotes the z-axis feed rate, and the C-axis speed in terms of n (rpm) takes the following form:

(14) $$\left\{\begin{array}{c} \alpha \left(t\right)=\frac{2\pi \mathrm{nt}}{60},\\ \;\theta (t)=\mathrm{mod}(\alpha ,2\pi )\;.\; \end{array}\right.\;$$

### 3.2. Tuning of System Servo-Control System

**Figure 11:**
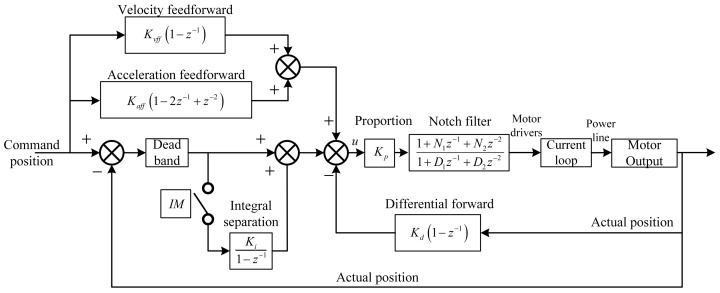
Diagram of the servo control system.

The control block diagram of the servo system is presented in Figure 11. The servo system mainly includes a position loop and a current loop. The velocity loop can improve the damping characteristics and stability but will lead to hysteresis. The fast response-ability is more important for STS machining, so the velocity loop is no longer used in this experiment. The position loop is realized by the universal motion and automation controller (UMAC) to achieve high-precision positioning. UMAC has the capacity to control 32 axes at the same time. We chose the same control structure for every linear and rotary axis out of convenience. The current loop is implemented in a driver to reduce the current fluctuation and ensure accurate torque output. For more information on this control block diagram, please refer to the UMAC manual from the Delta Tau Company.

The UMAC improves the traditional proportional-integral-differential (PID) algorithm and provides a PID servo control strategy based on the combination of feedback correction and feedforward compensation, as demonstrated in Figure 11. This essentially includes integral separation, differential forward, feedforward compensation, and notch filtering. The feedforward compensation chiefly lessens the tracking error caused by factors such as speed change, system inertia, and friction. The feedforward parameters include velocity feedforward K_vff_ and acceleration feedforward K_aff_. The factor K_vff_ adds an amount to the control output proportional to the desired velocity of the motor. It is intended to reduce the tracking error due to the damping introduced by differential K_d_, analog tachometer feedback, or physical damping effects. The factor K_aff_ adds an amount to the control output proportional to the desired acceleration for the motor. It is intended to reduce the tracking error due to inertial lag.

**Figure 12:**
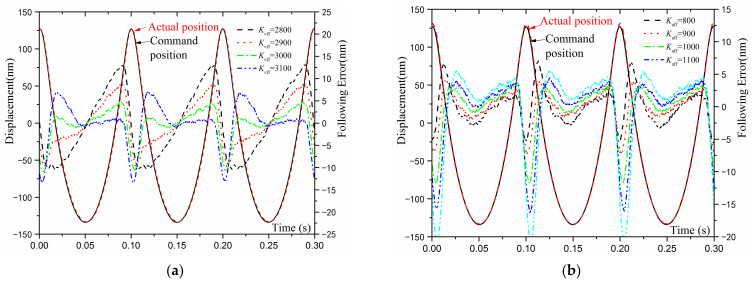
The tracking capability before and after the feedforward parameters adjustment: (**a**) Effect of K_vff_ on FE, (**b**) Effect of K_aff_ on FE.

The higher the machine frequency, the faster the feed axis speed and the greater the acceleration, and the larger the tracking error is, resulting in a larger discrepancy between the required and actual running compensation position. Under various machining paths and machining frequencies, the minimum tracking error of the feed axis should be guaranteed. As mentioned above, the factor K_vff_ tends to reduce the system delay caused by the damping effect of the differential elements, while K_aff_ has a tendency to lessen the following error (FE) due to the overshoot caused by the system inertia. It is necessary to adjust the velocity and acceleration feedforward parameters according to the actual motion command. Figure 12a exhibits the effect of K_vff_ on the tracking errors. The corresponding values of the plotted curves are 2800, 2900, 3000, and 3100. The actual position lags behind the command trajectory first and then leads the command trajectory as K_vff_ continues to increase. Therefore, the K_vff_ value associated with the smallest FE is taken for the subsequent analysis. Figure 12b presents the effect of K_aff_ on the tracking error. The K_aff_ values corresponding to the demonstrated curves are 800, 900, 1000, 1100, and 1200. Similar to the K_vff_ parameter, with the increase of K_aff_, the overall FE first reduces and then enlarges so that the K_aff_ value with the smallest FE is selected.

### 3.3. Experimental Results

**Figure 13:**
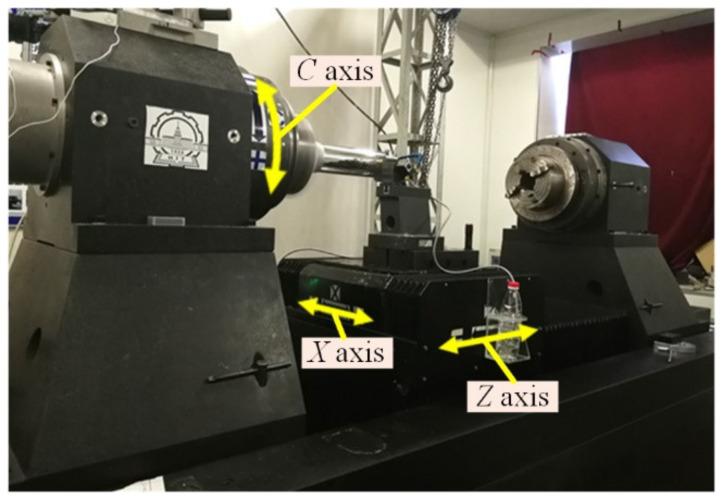
Cutting experiment of the mandrel roundness error compensation.

Firstly, the mandrel made of 6061-aluminum alloy is acceptably cut. The X-directional depth-of-cut of the cutting tool is kept constant (2 μm), and the spindle rotates with a speed of 300 rpm so that a round surface can be generated. The cutting tool is a round-nose single-crystal diamond tool with a nose radius of 0.95 mm, a rake angle of 0°, and a clearance angle of 7°. Figure 13 demonstrates the actual cutting process.

**Figure 14:**
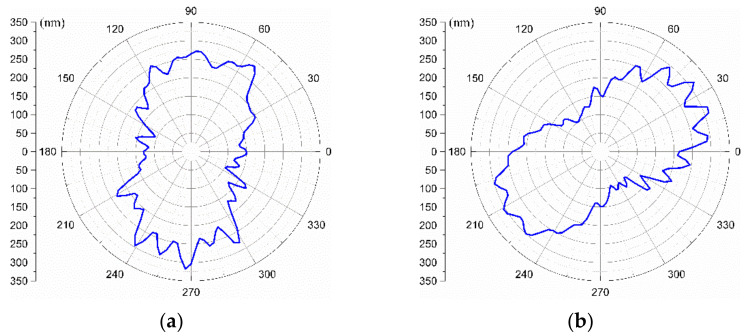
Radial error motion of the spindle separated from the WRE: (**a**) SREM along OX-axis, (**b**) SREM along OY-axis.

After machining and following the procedure mentioned above, the SREM and WRE are separated through the three-point method. The obtained results in Figure 14 reveal the SREM separated from the WRE, which is plotted in a polar coordinate system. As can be seen in Figure 14, the spindle motion error is approximately 0.2 μm along OX-axis. (Please note that the minimum radius is not zero in order to make the cross-sectional shape clearer in the figure). It can also be intuitively observed that the SREM has a 2 up (undulations per revolution) major component.

**Figure 15:**
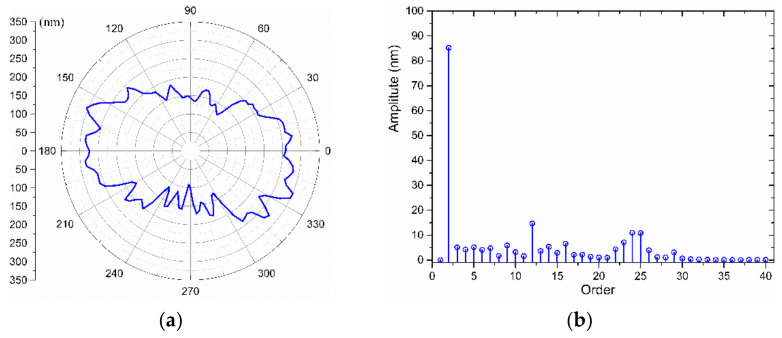
The WRE of the mandrel and its spectrum: (**a**) WRE graph in a polar coordinate system, (**b**) Error spectrum.

The polar and spectrum diagrams of the WRE have been illustrated in Figure 15. As can be seen from the roundness error shape of Figure 14 and Figure 15, the main source of the WRE is the SREM, and the shapes of the WRE and SREM are roughly complementary, indicating that the random error ε(θ) in Equation (13) is quite small and stable. This issue reflects the side that the asynchronous error of the air bearing is small. The WRE is 214.87 nm, as evaluated by the LCC method, and 199.19 nm by the MZC method.

**Figure 16:**
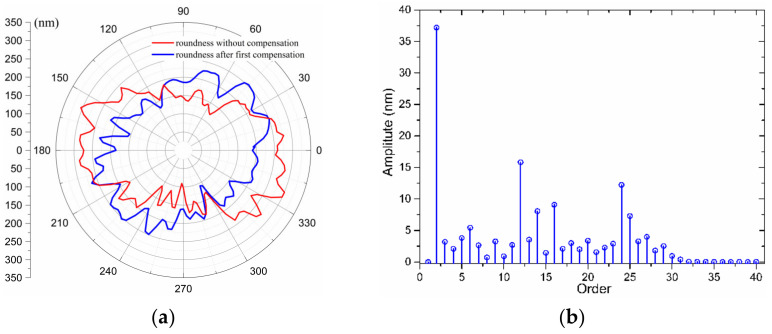
The WRE of the mandrel and its spectrum after the first compensation: (**a**) WRE graph in a polar coordinate system, (**b**) Error spectrum.

In order to achieve the required 0.1 μm out-of-roundness on the workpiece surface, a compensation process of the error motions based on the measurement results is necessary. Following the data process above, the compensation data are generated. The movement of the X-slide and the rotation of the spindle are synchronized to perform the STS technique. After the cutting process, the WRE is measured again. Figure 16 illustrates the roundness error polar diagram and spectrum of the measured section after the first compensation. The predicted WRE by the LCC approach is 109.06 nm, and by the MCC method is 101.37 nm. The plotted results in Figure 16 reveal that the low-order components of the error are substantially decreased after compensation, and the roundness error is reduced by about 50%, which shows the feasibility of compensating for the radial motion of the spindle.

**Figure 17:**
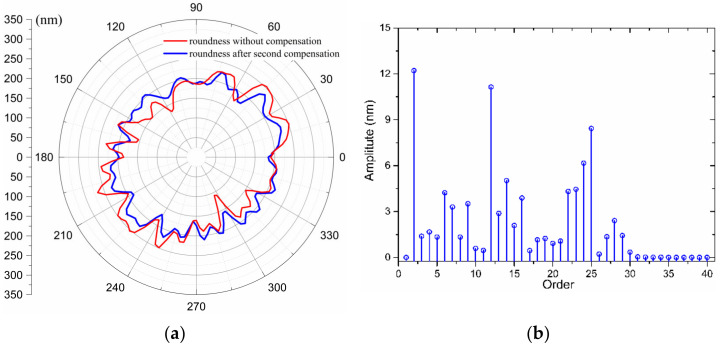
WRE of the mandrel and its spectrum after second compensation: (**a**) WRE graph in a polar coordinate system, (**b**) Error spectrum.

Although the roundness error after the first compensation is obviously reduced, it is not entirely eliminated. One possibility that could cause error residuals is that it is not rigid from the motor drive point to the tool holder, and there is a certain delay. In order to further enhance the roundness, the workpiece is processed with error compensation again, and the compensation tool path is superimposed with the previous one. The polar coordinate diagram of the section contour error after error compensation has been illustrated in Figure 17. The WRE is 80.11 nm, as evaluated by the LCC method, and 70.98 nm by the MZC method. The demonstrated results in Figure 17 display that the low-order components of the error are remarkably reduced after compensation, but the total roundness error is reduced to less than 40% compared to the case of no compensation. In Figure 17, in addition to the 2-cycle error at a low frequency, the 12-cycle error is also very obvious, which corresponds to the number of pole pairs of the spindle motor. Obviously, the magnetic pull of the motor affects the machining and measuring process.

**Figure 18:**
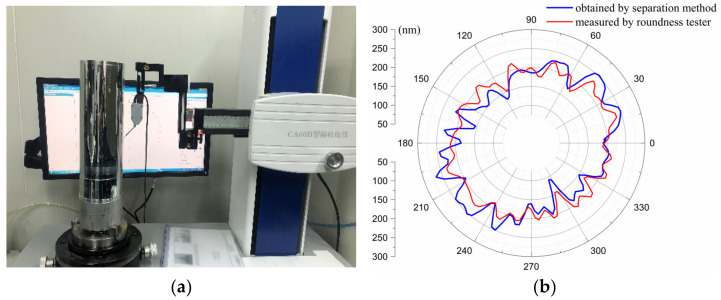
WRE of the mandrel tested by the in situ separation method and roundness tester: (**a**) WRE tested by a roundness tester, (**b**) WRE measured by various methods.

Further experiments reveal that increasing the number of compensation iterations cannot further decrease the error. This fact indicates that the compensation approach using the STS is limited and cannot compensate for all errors. There are not only the errors introduced by the measurement but also the reasons for the poor dynamic response capability of the system. As illustrated in Figure 12, the existing servo system has a tracking error during the tracking of the error compensation curve. In order to exclude the error introduced by the measurement, the WRE, after cutting the compensation, is measured with a roundness meter, as demonstrated in Figure 18. Typically, the roundness and waviness of the cylindrical parts are characterized using two main methods: a radius change method, also called a non-reference method, and a V-block method, known as a reference method [31]. The most reliable measurement method for the roundness is a roundness meter. The WRE is 75.32 nm, as evaluated by the LCC method. The measured error of these two methodologies is less than 5 nm, indicating that the three-point separation method is correct and effective. Compared with the error obtained by the three-point separation method, the results measured by the roundness meter are generally smoother.

## 4. Discussion

The experiment shows that it is possible to machine a large mandrel with a roundness that is less than 0.1 um through in situ compensation. One key to achieving this high accuracy is to compensate two more times, which shows that the dynamic process should be modeled more accurately, and the remaining error may decrease further. 

Compared with the traditional FTS process, the use of the STS method compensating the spindle error does not require a complex measurement and control system; this research is also beneficial and can be further applied to the error control of cutting complex aspherical surfaces. For the further improvement of the roundness of the workpiece surface, it is necessary to compensate for the high-frequency error of the diamond turning machine, where a fast-tool-servo (FTS) unit is needed. The aerostatic spindle not only has a radial motion error but also an angular motion error, which results in various radial motion errors with different positions on the z-axis; therefore, corresponding measurements and compensation methods are required to enhance the cylindricity of the workpiece surface.

## 5. Conclusions

The radial error motions of a large diamond turning machine used for the fabrication of the precision mandrel were measured and compensated. Three high-precision capacitance displacement sensors and corresponding time-domain three-point error separation techniques were employed to detect the cross-section of the workpiece and separate the roundness error from the rotation motion error. It was verified that the aerostatic spindle has a radial motion error of 0.2 μm, which leads to approximately the same roundness error of the workpiece. The main component of the roundness error was reported to be a double-fundamental low-frequency harmonic error, which was reversed and integrated into the depth-of-cut data of the STS process for compensation. With the compensation of the error motions, the out-of-roundness of the workpiece surface was reduced to less than 0.1 μm, which meets the requirement for the large mandrel. 

## Data Availability

The data supporting the reported results by the authors can be sent by e-mail.
